# Comprehensive evaluation of cross cancer generalization in histopathology segmentation models across 21 tumor types

**DOI:** 10.1038/s43856-026-01601-x

**Published:** 2026-05-05

**Authors:** Tillmann Bedau, Christian Harder, Abdulkader Al-Shughri, Yuan Wang, Alexey Pryalukhin, Marie-Lisa Eich, Su Ir Lyu, Reinhard Büttner, Alexander Quaas, Yuri Tolkach

**Affiliations:** 1 Institute of Pathology, University Hospital Cologne, Cologne, Germany; Medical Faculty, University of Cologne, Cologne, Germany; 2 Institute of Pathology, University Hospital Wiener Neustadt, Danube Private University, Wiener Neustadt, Austria; 3 Institute of Pathology, Charité – Universitätsmedizin Berlin, corporate member of Freie Universität Berlin, Humboldt-Universität zu Berlin and Berlin Institute of Health, Berlin, Germany

**Keywords:** Cancer, Computational biology and bioinformatics

## Abstract

**Background:**

Artificial intelligence has significantly advanced computational pathology by enabling high-resolution, clinical-grade tumor segmentation models with state-of-the-art diagnostic accuracy. Creating such models is resource-intensive, requiring substantial time and domain expertise. Additionally, deep learning models are typically restricted to single tumor types, making it challenging to develop separate models for each tumor type. Cross-cancer generalization of segmentation models could address this bottleneck and pave the way for pan-cancer segmentation models.

**Methods:**

We evaluated the cross-tumor generalization capability of five tissue segmentation models (breast, colon, lung, kidney, prostate) using 21 cancer types from The Cancer Genome Atlas, totaling over 7,700 whole slide images. Representative large tumor and benign regions were manually selected, and segmentation accuracy was evaluated using a semiquantitative scale (0-10).

**Results:**

Here we show that the lung model demonstrates excellent cross-cancer performance (overall mean score 7.9 ± 2.1), effectively segmenting tumor regions in many non-lung cancers with segmentation accuracy similar to its native domain in 11 of 19 other epithelial tumors and melanoma, achieving particularly strong results in ovarian cancer (9.2 ± 0.9). The breast and colon models also show strong cross-domain performance, while the kidney and prostate models exhibit more limited generalization. Overall, high-precision segmentation is achievable in most cancer types using existing models.

**Conclusions:**

Existing segmentation models generalize across multiple cancer types, reducing the need to develop new, entity-specific models from scratch. This cross-domain generalization enables fast-track model development and supports future creation of robust pan-cancer segmentation models. Leveraging these capabilities could accelerate clinical integration of pathology artificial intelligence tools and enable reproducible biomarker discovery.

## Abstract (web summary)

Bedau et al. evaluate cross-cancer generalization of five histopathology segmentation models across 21 tumor types comprising over 7,700 whole slide images. A model trained and validated on lung adenocarcinoma and squamous cell carcinoma achieves excellent segmentation in 11 of 19 non-native cancers, enabling development time reduction to under one week.

## Plain language summary

Computer tools can help doctors who examine tissue samples under the microscope by automatically marking areas of cancer. However, building these tools for each type of cancer takes a lot of time and specialist knowledge. We asked whether a tool built for one type of cancer could also work for other types. We tested five such tools, each originally built for a specific cancer (breast, bowel, lung, kidney, or prostate), on tissue images from 21 different cancer types, covering more than 7,700 images in total. We found that several tools, especially the one built for lung cancer, could accurately identify cancer regions in many other cancer types as well. This means that existing tools can be reused for new cancers without having to build them from scratch, cutting the time needed from months to days and helping bring these computer-assisted tools to hospital laboratories sooner.

## Introduction

The field of computational pathology has seen remarkable advancements in recent years, with artificial intelligence (AI) models, mostly deep learning (DL)-based, increasingly demonstrating their potential to complement traditional histopathological analysis.^1–3^ However, certain limitations of DL models constrain their broad application, with only few clinical-grade tools available on the market. One of the major shortcomings of DL models, particularly in a diagnostic oncopathology domain (e.g., multi-class tissue segmentation, tumor detection, grading, subtyping, etc.), is their restriction to one domain, i.e., to one tumor type.^4^ Given the large number of malignant tumor types,^5^ more than 30 different models would need to be trained just for the initial processing of histological slides (e.g., patch-level classification or multi-class tissue segmentation). This is not to mention the additional models required for each subsequent task (such as grading, subtyping, prognosis, etc.). Notably, another significant bottleneck of modern clinical-grade DL models, resulting from considerations mentioned above, is the need for thorough clinical validation, which includes multi-institutional data and can take at least 1–2 years per model.

A particularly active area of research has been the development of large histopathology foundation models,^6–10^, which provide feature embeddings for downstream deep learning tasks.^11^ These models, often combined with weakly supervised training approaches, do not require extensive manual annotations by domain experts, making them more accessible and easier to scale.^12^ While these models are valuable for predicting slide-level or patient-level labels like tumor presence, subtype, or therapy response,^2^ their patch-level (not pixel-level) resolution is often inadequate in providing the precise spatial information required by pathologists to accurately localize tissue features (see “Discussion”). This architectural constraint—rather than the training paradigm itself—limits their direct application to pixel-wise semantic segmentation tasks. In contrast, segmentation models, though more demanding in terms of annotation, offer pixel-level precision in delineating tissue structures. Pixel-wise semantic segmentation has become the state-of-the-art approach for diagnostic pathology models, reflecting how pathologists visually analyze tissue: not in discrete square regions, but by following precise boundaries of different structures. This precision not only facilitates quantitative analyses that exceed human capabilities but also enhances the explainability of AI-driven insights,^13^ making them more useful and trustworthy for pathologists to integrate into clinical workflows. Furthermore, pixel-level segmentation enables critical downstream applications, including quantitative biomarker extraction (e.g., tumor-stroma ratio, tumor budding assessment, immune cell quantification within defined tissue compartments), precise tissue masking for single-cell analysis pipelines, and integration with emerging spatial omics technologies (spatial transcriptomics/proteomics) that require accurate tissue boundaries for region-specific molecular analysis. In a recent study,^14^ we could show that even for a simple task such as lung cancer subtyping (adenocarcinoma vs. squamous cell carcinoma), fully supervised models outperform weakly-supervised approaches building upon histopathology foundation models or a combination of foundation model and the CLAM^15^ approach. With increasing computing power, diseases are now studied at ever-increasing levels of detail, from tissue structures down to individual cells. Studying cellular compositions using single-cell detection and classification models requires precisely pre-segmented tissue structures.^16^

Recent advances in general-purpose segmentation include the Segment Anything Model (SAM)^17^ and its successor SAM2,^18^ with medical adaptations MedSAM^19^ and MedSAM2,^20^ as well as BiomedParse,^21^ a foundation model for joint segmentation, detection, and recognition across nine imaging modalities. SAM, SAM 2, MedSAM, and MedSAM2 require user-provided prompts (points, boxes, or masks) to specify segmentation targets. BiomedParse supports both text-prompted segmentation and automatic object recognition. However, these models focus on identifying and delineating individual objects, whereas our models perform dense multi-class semantic segmentation, assigning every pixel to predefined tissue classes—essential for quantitative pathology workflows requiring consistent tissue compartment quantification.

We have previously developed high-performance segmentation models for specific cancer types, including colon,^22^ lung,^14^ breast, kidney, and prostate cancers.^23,24^ These models have demonstrated excellent accuracy within their respective domains, but their potential for cross-entity generalization—applying them to disease entities that were not part of their original training—remains largely unexplored. Exploring this potential could significantly reduce the time and resources required to develop new, entity-specific models. Furthermore, this line of research opens the possibility of creating a versatile segmentation model, similar to the foundation models discussed earlier, capable of accurately segmenting any tissue type across various disease entities.

In this study, we evaluate the cross-cancer generalization capabilities of our previously developed segmentation models across a wide range of cancer types. We utilize the extensive and publicly available resources of The Cancer Genome Atlas (TCGA) to derive a cohort comprising 21 distinct cancer projects and over 7700 whole slide images. By applying our five cancer-specific models to this broad dataset and systematically scoring their performance, we identify that for the majority of cancer types, high-precision segmentation of tumor regions (and benign tissue) is possible using previously developed models. This opens a possibility for fast-track single tumor model training^23,25^ and for the development of a pan-cancer foundational segmentation model in the future. We propose several alternative strategies how cross-tumor generalizability can be leveraged to develop precise clinical-grade segmentation models; these could offer more efficient pathways compared to traditional, time-intensive training approaches. We are making our pan-tumor dataset with manual tumor segmentation scorings available to the research community.

## Methods

### Cohort selection and image acquisition

A comprehensive cohort from The Cancer Genome Atlas (TCGA) was selected, consisting of 21 distinct cancer projects. The specific TCGA projects included are BLCA (bladder cancer), BRCA (breast cancer), CESC (cervical squamous cell carcinoma), CHOL (cholangiocarcinoma), COAD + READ (colon and rectum adenocarcinoma), ESCA (esophageal cancer: adenocarcinoma and squamous cell carcinoma), HNSC (head-and-neck squamous cell carcinoma), KICH (chromophobe renal cell carcinoma), KIRC (clear-cell renal cell carcinoma), KIRP (papillary renal cell carcinoma), LIHC (liver hepatocellular carcinoma), LUAD (lung adenocarcinoma), LUSC (lung squamous cell carcinoma), MESO (mesothelioma), OV (ovarian carcinoma), PAAD (pancreatic adenocarcinoma), PRAD (prostate adenocarcinoma), SKCM (skin cutaneous melanoma), STAD (stomach adenocarcinoma), THCA (thyroid carcinoma), and UCEC (uterine corpus endometrial carcinoma), totaling 7706 whole slide images (WSIs). We included mostly carcinomas (and malignant melanoma) and excluded other tumor entities such as soft tissue and bone tumors (sarcomas), neoplasms of the nervous system, germ cell tumors, and hematological neoplasms. The selection criteria for these slide images were: (1) non-frozen tissue section, (2) adequate tissue section/digitization quality, at least focally (see selection of regions of interest below). All available SVS image files for each project (excluding frozen sections) were downloaded using the GDC Data Transfer Tool.^26^ Digitization was performed in the course of the TCGA project by the scanner family of Leica Biosystems (Wetzlar, Germany; models AT2, CS2, etc.; not GT450) with most slides scanned under ×40 magnification (few slides under ×20 magnification) and micron per pixel (MPP) parameter of approximately 0.25.

### Ethics

This study was approved by the Ethics Committee of the Medical Faculty of the University of Cologne (reference numbers 22-1233 and 20-1583) and was conducted in accordance with the Declaration of Helsinki. All data used in this study were obtained from The Cancer Genome Atlas (TCGA), a publicly available and de-identified dataset. TCGA participants provided informed consent under the protocols of their respective contributing institutions as part of the original TCGA program. No additional patient consent was required for this secondary analysis of de-identified, open-access data, and no proprietary data or data from patients of the authors’ institutions were used.

### Image preparation for analysis

All WSI files were imported into QuPath v0.5.0^27–30^ for image preprocessing. A custom Groovy script was used to create two rectangle annotations per WSI: one for tumor tissue and one for benign tissue. Each annotation was set to a size of 12,500 × 10,000 pixels representing a sufficiently large region capturing significant portions of tumor or benign tissue from a morphological point of view. A pathologist manually dragged these rectangular annotations to representative tumor and benign regions of the slide, if available. These regions of interest (ROIs), with a maximum of two per slide (one for tumor and one for benign), were then exported as JPG files using another Groovy script for further analysis with a typical compression (=usual digitization compression, 80%, as utilized by most histoscanners). The files were sorted by tumor type to allow further analysis.

### Development of the breast model

The breast cancer algorithm was developed using hematoxylin & eosin stained (H&E) whole-slide images (WSIs) from the BRCA cohort of the The Cancer Genome Atlas (WSI *n* = 274). In most cases, for training we took one slide per patient case (patient *n* = 270). All cases were selected by an expert pathologist (YT) to assure the representative nature of tumor morphologies, including different histological subtypes, such as invasive-ductal (NOS), invasive-lobular, mucinous, micropapillary, etc. TCGA-TRAIN and UKK cohorts were extensively manually annotated by expert pathologists (AAS, YT) in QuPath software version v.0.3.2 and later. All annotations were performed using “dense” annotations strategy in most slides (all pixels annotated in selected regions) and using high-precision (e.g., single cell annotations in invasive-lobular subtype). During this process, we selected 3–5 representative regions in each slide. The final version of the dataset contained 106,546 single patches and 12 classes (11 tissue classes and background). Algorithm training was performed using a previously developed pipeline.^14,22^ Briefly, we trained a semantic segmentation neural network. Encoder and decoder architectures were considered as hyperparameters with U-Net and U-Net++ used for later and variations of EfficientNetB convolutional neural networks for former. Batch size, learning rate, patch size, and magnification were all tested as a hyperparameter range. In all trainings, we used data augmentation (flips, rotations of the image, jpeg compression, Gaussian noise, hue/saturation/contrast/brightness in a random mode) and batch engineering, with the latter ensuring that each batch contains equal number of patches of each class. We also used oversampling of all underrepresented classes at patch level during training with oversampling target being tumor class as the most represented and relevant in our dataset. A small validation subset (ca. 10%) was reserved from TCGA-TRAIN for validation/fine-tuning purposes. The final algorithm was trained using UNet++ decoder, EfficientNetB0 encoder, resolution of 1.0 micron-per-pixel, patch size of 512 px, batch size of 24, and cross-entropy weighted loss function. The algorithm was extensively validated using independent datasets showing high segmentation accuracies (Dice score >0.85).

### Development of the kidney model

The TCGA kidney cancer cohort was used for algorithm development (clear-cell/KIRC, papillary/KIRP, and chromophobe/KICH subtypes). Manual annotations were performed by expert analysts using similar principles as described for the breast algorithm. The total number of annotated images was 131 for KIRC, 244 for KIRP, and 55 for KICH. The trained version of the algorithms has the same architecture as described for breast cancer and detects 10 classes and background. The final model was extensively validated using three independent datasets showing high segmentation accuracies (Dice score > 0.941).

### Multi-class tissue segmentation using trained models

No new models were trained in this study; we exclusively evaluated the cross-cancer generalization of five previously developed segmentation models. All exported JPG files were used as input for a Python-based pipeline that implements patch-based pixel-wise segmentation of the ROIs (Python v3.12.2). This pipeline generates masks with pixel-wise class coding and, based on these, creates colored segmentation masks for each ROI, with distinct colors representing different tissue classes (color-class mappings outlined in Supplementary Table 1). Overlay images were produced for visual evaluation by pathologists, by superimposing the colored segmentation masks onto the original H&E-stained ROIs.

The segmentation process was conducted using five previously developed segmentation models, each specific to breast, colon, lung, kidney, and prostate tissues. The lung, colorectal and prostate models were previously published.^14,22,24^ The summary for the development of the breast and kidney models is provided above. All five models share a common architecture: UNet++ decoder with EfficientNet encoders (EfficientNetB0 or B1), implemented in PyTorch using the segmentation_models_pytorch package (v0.2.1 for the colon model, v0.3.1 for all other models) [43]. Models operate on 512 × 512-pixel tiles at 1.0 µm/pixel resolution (approximately ×10 magnification) and were trained for pixel-wise multi-class tissue segmentation with 10–13 tissue classes (prostate: 5 classes). The tissue classes were selected based on their relevance in routine diagnostic pathology and include: tumor epithelium (invasive carcinoma and, where applicable, in situ neoplasia such as DCIS/LCIS in breast or high-grade adenoma in colon), tumor stroma, necrosis, extracellular mucin, lymphoid tissue and inflammatory infiltrates, benign stroma (including fat, vessels, nerves, and muscle), blood, and organ-specific benign epithelial structures (e.g., benign breast lobules, colonic mucosa, bronchial epithelium, renal parenchyma). These classes enable quantitative assessment of diagnostically important tissue compartments, such as tumor-stroma ratio calculation, necrosis quantification, and precise delineation of the tumor microenvironment. Training datasets were derived from TCGA whole-slide images with dense manual annotations by expert pathologists in QuPath. For each model, training utilized approximately 20–30% of available images from the respective TCGA project(s). Notably, each TCGA project comprises cases from up to 36 different pathology institutions with varying laboratory practices (fixation, sectioning, staining) and digitization equipment, representing a heterogeneous multi-institutional training cohort. All models were trained using oversampling of underrepresented tissue classes and batch-level class balancing. Data augmentation included geometric transformations (flips, rotations) and color augmentation (brightness, contrast, gamma, hue, saturation); additional augmentations varied by model (see original publications and Suppl. Methods). No stain normalization was used during training or inference. Encoders were fully retrained from ImageNet initialization. A validation subset (approximately 10%) was reserved for hyperparameter tuning. All models achieved Dice scores >0.85 on validation data and were extensively validated on independent, multi-institutional external cohorts demonstrating robust generalization across different scanners and institutions.

No stain normalization or additional preprocessing was used for inference. The models were applied sequentially to the ROIs from single malignant tumor types. The final overlays were saved as JPG files and masks with class coding as PNG files. JPG overlays were downsampled to a size of 2500 × 2000 pixels for further evaluation by pathologists.

### Scoring of segmentation outputs: software development and implementation

A custom browser-based application was developed to facilitate the scoring of segmentation outputs. JPG overlay images were first converted to Deep Zoom Image (DZI) format using libvips v8.15.1.^31^ A FastAPI server (v0.108.0)^32^ was then used to serve these images to a web frontend, where they were displayed using OpenSeadragon v4.1.0,^33^ allowing for smooth navigation and zooming of images. The custom web application facilitates the scoring process by displaying the outputs of all five models side by side, with an option to dynamically score each image individually (Supplementary Fig. 1; software code is publicly released). Scoring data is stored in an SQLite database and can be easily navigated and exported as a CSV file for final analysis.

Each segmentation output (five per each ROI, corresponding to the number of models) was evaluated by a trained resident pathologist (TB) controlled by attending physician with extensive surgical pathology experience (YT) on a semiquantitative scale from 0 to 10, where 0 represents the worst segmentation quality and 10 represents ideal tumor/stroma segmentation. The criteria for scoring included the accuracy of tumor/stroma identification, precision of object boundaries, and the number of tumor structures missed. Scores were assigned as follows: 10 for “ideal” segmentations, 8 for “excellent”, 6 for “good”, 4 for “poor”, 2 for “very poor”, and 0 for “unacceptable” (see Fig. 2). Difficult cases were discussed resulting in a consensus score.

### Validation of manual scoring system

We assessed inter-rater reliability by having a board-certified pathologist (YT) independently score a subset of 598 segmentation outputs across all five models. Intraclass correlation coefficient (ICC) was calculated using a two-way random effects model for consistency (single measures) with the “irr” package (v0.84.1) in R. Spearman’s rank correlation coefficient (ρ) was also computed to assess the monotonic relationship between scorers.

For quantitative validation, we performed Dice coefficient analysis on a separate subset of 346 ROIs from our own model training cohorts, where ground truth annotations were available. These cohorts included breast (*n* = 27 ROIs), colorectal (*n* = 134 ROIs), lung (*n* = 129 ROIs), and prostate (*n* = 56 ROIs) cancers.^14,22–24^ We exported both H&E ROIs and manual annotations from QuPath. For simplicity, we only exported tumor and tumor stroma annotations, with all other annotations being treated as background class.

When running inference with the different models, we mapped the model outputs to the classes used in the ground truth annotations exported from QuPath. For all models, various epithelial classes were mapped to the tumor class, stroma-related classes were mapped to the tumor stroma class, and all remaining classes were mapped to the background class. Specifically:

For the prostate model (*n* = 4 classes + background class), tumor and benign glands were mapped to tumor, and benign stroma to tumor stroma. For the breast model (*n* = 13 classes + background class), tumor, DCIS, LCIS, skin, and benign epithelium were mapped to tumor, while tumor stroma and stroma were mapped to tumor stroma. For the lung model (*n* = 12 classes + background class), tumor, bronchus, and bronchial glands were mapped to tumor, while tumor stroma and stroma were mapped to tumor stroma. For the colon model (*n* = 11 classes + background class), tumor/high-grade adenoma was mapped to tumor, and tumor stroma to tumor stroma

For the prostate cohort, the ground truth annotations in QuPath were only available at a coarser (“regional”) level. The exported annotation/class maps were then converted into finer (“gland-level”) annotation maps using our previously published approach using the PESO algorithm.^23^

Dice coefficients were calculated by comparing the model predictions to expert-annotated ground truth tumor and stroma regions using the formula: Dice = 2|X∩Y|/(|X| + |Y|), where X represents the predicted segmentation and Y represents the ground truth annotation. During Dice score calculation, only the areas with non-background values in the ground truth annotations were included in the calculation.

The relationship between manual scores and Dice coefficients was assessed using ICC and a proportional agreement measure. For the latter, we calculated the percentage of cases where the scaled manual score (converted from 0–10 to a 0–1 scale) and Dice coefficient differed by no more than ±0.15. This “±15%“ metric provides a direct measure of agreement between subjective manual scores and objective Dice values, particularly useful for assessing concordance in regions with clustered scores. ICC was calculated using a two-way random effects model for consistency (single measures).

### Statistics and reproducibility

The study cohort comprised 7706 whole slide images from 21 TCGA cancer projects, from which 7616 tumor ROIs and 3296 benign ROIs were extracted and analyzed. Each ROI was processed by all five segmentation models, generating a total of 54,560 segmentation outputs for evaluation.

Inter-rater reliability was assessed on a subset of 598 segmentation outputs independently scored by two pathologists. Agreement was quantified using intraclass correlation coefficient (ICC; two-way random effects model, single measures, consistency) calculated with the “irr” package (v0.84.1) in R, and Spearman’s rank correlation coefficient (ρ). Quantitative validation against ground truth annotations was performed on 346 ROIs from source cohorts (breast *n* = 27, colorectal *n* = 134, lung *n* = 129, prostate *n* = 56) using Dice coefficients. The relationship between manual scores and Dice coefficients was assessed using ICC and proportional agreement (percentage of cases where scaled manual score and Dice coefficient differed by ≤0.15).

Descriptive statistics and data visualization were performed in R (v4.4.1) using the Tidyverse (v2.0.0), gtsummary (v1.7.2), and ggplot2 (v3.5.1) packages.

No sample size calculation was performed; the study utilized all available and qualifying TCGA images. No technical replicates were performed as each ROI was scored once per model; however, inter-rater reliability assessment on the 598-output subset confirmed scoring reproducibility (ICC = 0.87; 95% CI: 0.85–0.89). All scoring was performed by a trained resident pathologist (TB) with supervision by a board-certified pathologist (YT); difficult cases were resolved by consensus discussion.

### Use of generative AI

During the preparation of this work, ChatGPT 4o was used for language editing purposes and code improvement. After using this tool, all content and code was thoroughly reviewed and edited as needed, and the authors take full responsibility for the content and code of the published article.

## Results

### Preparation of pan-tumor dataset and generalizability testing

**Fig. 1:**
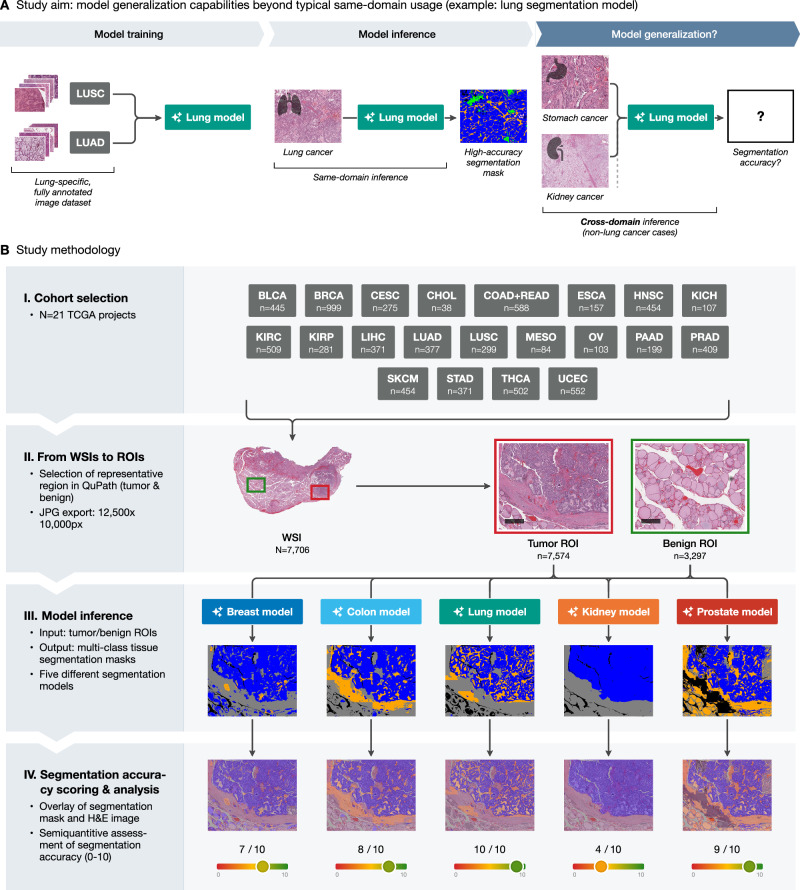
Study aim and methodology. **A** Typically, segmentation models are trained on data from a single tissue type and perform inference on the same type (lung tissue and lung model used as an example). This study investigates model performance when applied to tissue from entirely different origins. LUSC lung squamous cell carcinoma, LUAD lung adenocarcinoma. **B** A total of 7706 whole slide images (WSIs) from 21 distinct TCGA projects were selected. Representative tumor and benign regions of interest (ROI) were annotated in QuPath and exported. Five segmentation models (breast, colon, lung, kidney, prostate) were applied to the selected ROIs, producing multi-class tissue segmentation masks and overlays (blue: tumor tissue; yellow: tumor stroma; gray: other classes), which were manually scored on a scale from 0-10 and further analyzed. Scale bars: 500 μm.

During algorithm development, the main challenge is the precise pixel-level annotation of tumor regions, where epithelial tumor structures are often numerous, small, and have complex shapes (might take 2–5 h/case even for very small regions). In contrast, annotation of benign regions is typically quicker, often taking just a few minutes. The main aim of the study is to evaluate the cross-domain generalization capabilities of multi-class tissue segmentation models developed for one tumor type across a broad range of different malignant tumors (Fig. 1A). This allows them to be used for precise, time-saving pre-annotations in new tumors or to directly apply them to new tumors without re-training. To evaluate the generalization capabilities, we take a cohort of 7706 cases (*n* = 21 malignant tumors), resulting in the generation of 7616 large, manually annotated tumor ROIs and 3296 benign ROIs, representative of single case morphology (Fig. 1B). All ROIs were analyzed by five previously developed segmentation models, each trained on a specific cancer type: breast, colon, lung, kidney, and prostate. The segmentation models produced multi-class segmentation masks, overlaid on the original H&E images, with each color representing a distinct tissue type, such as tumor tissue or tumor stroma.

**Fig. 2:**
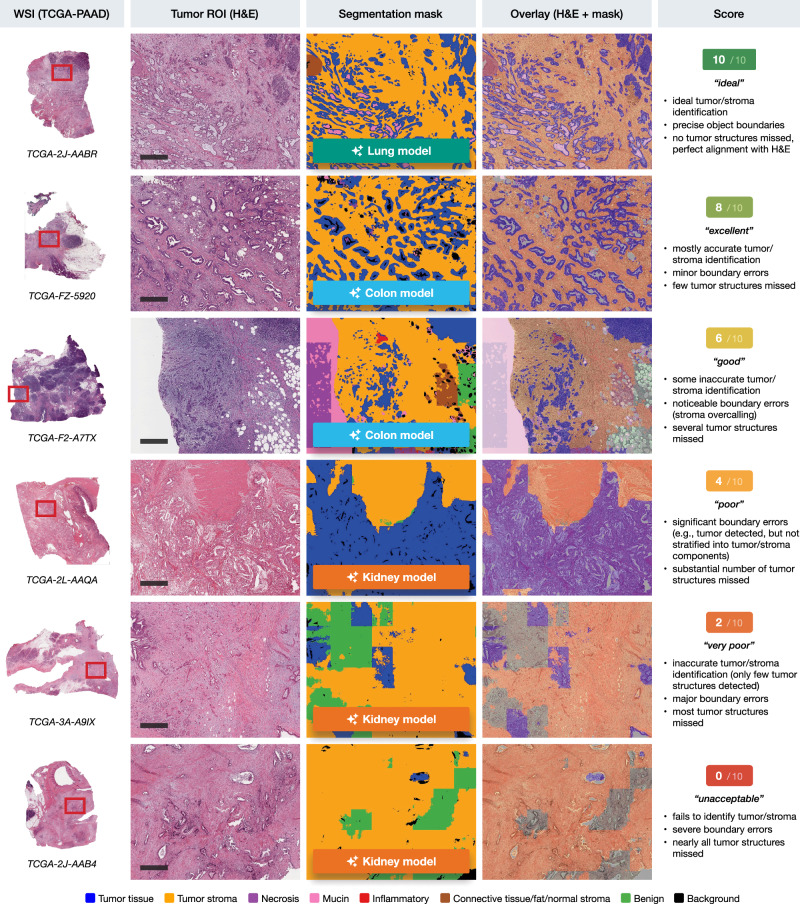
Representative tumor/stroma segmentations across a scoring range of 0 to 10 (TCGA-PAAD cases). Example whole slide images (WSIs) from the TCGA-PAAD (pancreatic adenocarcinoma) cohort with corresponding manually defined tumor regions of interest (ROIs), segmentation masks, and overlay images generated by different models (lung, colon, kidney). Scoring categories are illustrated from “ideal” (10/10) to “unacceptable” (0/10) based on tumor/stroma identification accuracy, boundary precision, and the detection of tumor structures. Tumor tissue is shown in blue, tumor stroma in yellow, with additional classes such as necrosis, mucin, and inflammatory cells indicated by specific colors in the legend. Scale bars: 500 μm.

The segmentation outputs were then manually scored on a semiquantitative scale from 0 to 10 by a trained pathologist, assessing the accuracy of tumor/stroma identification, boundary precision, and the general detection of tumor structures. This scoring was performed using a custom-developed browser-based scoring application that allowed for side-by-side comparison of the five different model outputs for each case (Supplementary Fig. 1). Figure 2 shows representative tumor/stroma segmentations across a range of scores from 0 to 10. These examples demonstrate the varying levels of segmentation accuracy, with the highest scores reflecting precise tumor/stroma identification and boundary definition, and lower scores indicating significant errors in segmentation.

### Validation of manual scoring system

**Fig. 3:**
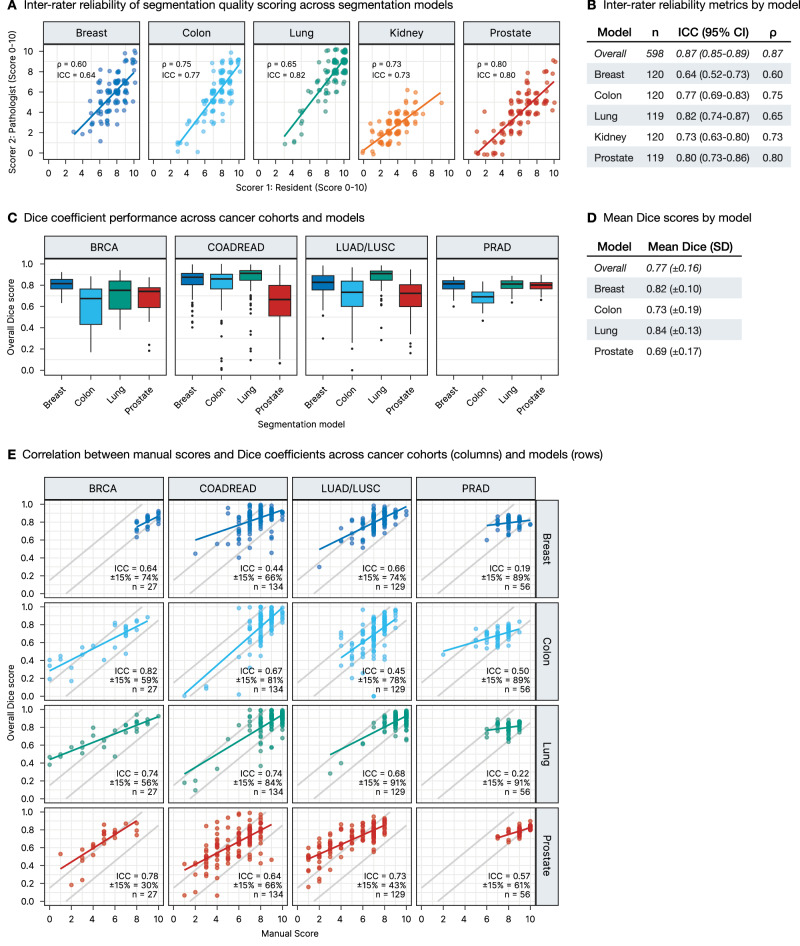
Validation of segmentation quality scoring methodology. **A** Inter-rater reliability across models: scatter plots of pathologist vs. resident scores with Spearman’s ρ and intraclass correlation coefficient (ICC) values for each model. **B** Inter-rater reliability metrics by model: sample size (*n*), ICC with confidence intervals, and Spearman’s *ρ*. **C** Dice coefficient performance across our own model training cohorts: breast cancer (BRCA), colorectal adenocarcinoma (COADREAD), lung adenocarcinoma and squamous cell carcinoma (LUAD/LUSC), and prostate cancer (PRAD) evaluated with different segmentation models. **D** Mean Dice scores with standard deviations by model. **E** Relationship between manual scores and Dice coefficients across our own model training cohorts and models. Gray lines show ±15% tolerance bands. Annotations display ICC values, percentage of points within tolerance bands, and sample sizes for each combination.

To address the inherent subjectivity of manual scoring, we implemented a dual validation approach (Fig. 3). First, we assessed inter-rater reliability through independent scoring by a board-certified pathologist on a subset of segmentation outputs (*n* = 598). The inter-rater correlation revealed excellent agreement (ICC = 0.87; 95% CI: 0.85–0.89, Spearman’s *ρ* = 0.87), with ICC values ranging from 0.64 to 0.82 and Spearman’s *ρ* values from 0.60 to 0.80 across individual segmentation models—with overall values exceeding individual ones due to distinct score distributions across models (Fig. 3A, B).

Second, we performed quantitative validation using Dice coefficient analysis against ground truth annotations from our source cohorts (breast, colorectal, lung, and prostate cancers, *n* = 346 ROIs). Dice coefficients were calculated by comparing model predictions to expert-annotated tumor and stroma regions. As shown in Fig. 3C and quantified in Fig. 3D, segmentation models achieved an overall mean Dice score of 0.77 (±0.16), with the lung model demonstrating the highest overall performance (0.84 ± 0.13), followed by the breast model (0.82 ± 0.10), while the Prostate model showed lower average performance (0.69 ± 0.17).

To validate our manual scoring approach, we then analyzed the relationship between these objective Dice coefficients and our manual scores of the source cohort segmentations, scored using the same methodology as our main TCGA dataset (Fig. 3E). We assessed this relationship using both ICC and a proportional agreement measure showing the percentage of cases where scaled manual scores and Dice coefficients differ by no more than ±15%. The ICC values ranged from 0.19 to 0.82 across model-cohort combinations. Notably, some combinations with clustered high-performance scores (e.g., breast and lung model on prostate cohort) showed relatively low ICC values (0.19–0.22) but demonstrated remarkably high proportional agreement (89–91% within ±15%), indicating strong concordance between manual and Dice-based assessments despite the restricted score range. Other combinations (e.g., lung model on lung cohort) showed both high ICC (0.68) and high proportional agreement (91% within ±15%). Crucially, since objective scoring on the entire TCGA dataset is not feasible, these results from our model training cohorts validate our manual scoring methodology, demonstrating that our approach effectively captures segmentation performance through both correlation and direct agreement measures. This validation supports the reliability of our approach for subsequent large-scale cross-domain generalization analyses.

### Very high, model-dependent levels of generalizability are evident across a broad range of malignant tumors

**Fig. 4:**
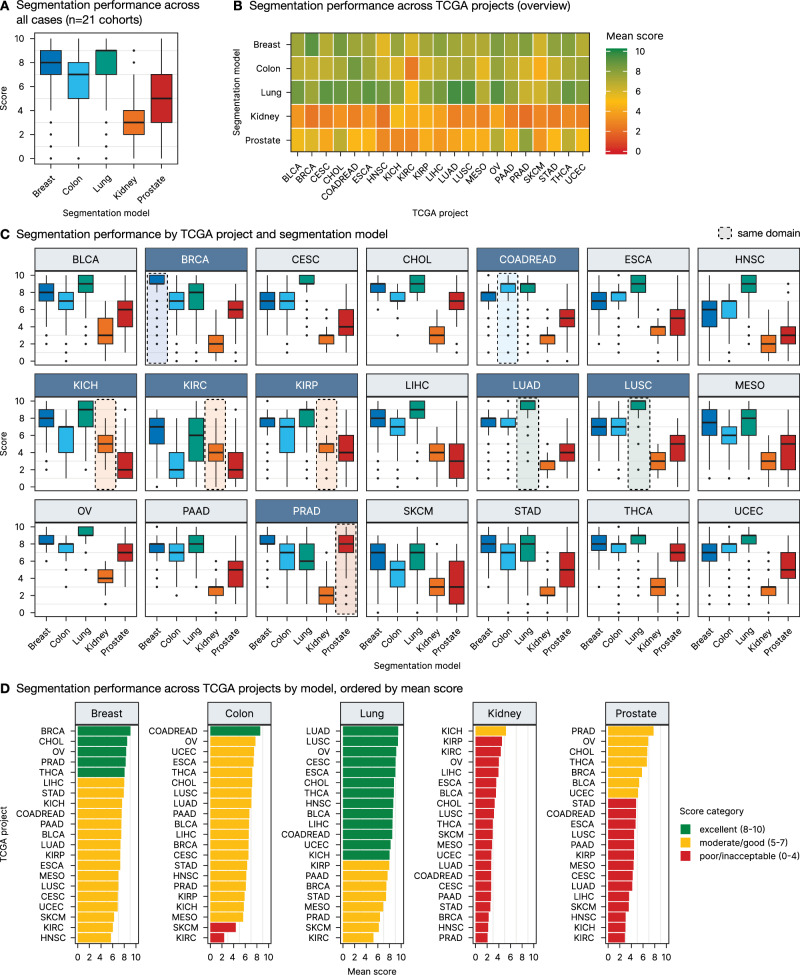
Quantitative analysis of segmentation performance across different models and TCGA projects. **A** Segmentation performance across all tumor ROIs for each model. **B** Overview of segmentation performance across TCGA projects by model, displayed as a heatmap. **C** Detailed segmentation performance by TCGA project and segmentation model. TCGA projects for which a native segmentation model is available are depicted in dark blue with the respective model performance emphasized in the boxplot. **D** Segmentation performance across TCGA projects by model, ordered by mean score. Scoring categories are color-coded: excellent (8–10), moderate/good (5–7), and poor/unacceptable (0–4).

The segmentation scoring results across all 21 TCGA projects demonstrate that, overall, the lung model exhibited the best performance with an overall mean score of 7.9 ± 2.1, while the kidney model showed the lowest performance with an overall mean score of 3.0 ± 1.4 across the different cancer types (Fig. 4A and Table 1). The lung model performed exceptionally well not only in its native cancer types, lung adenocarcinoma (LUAD) and lung squamous cell carcinoma (LUSC), but also generalized effectively to a wide range of non-lung cancer entities with an excellent segmentation accuracy of tumor regions (Fig. 4B). Similarly, the colon and breast models displayed strong performance across other cancer types besides their native colon and rectum adenocarcinoma (COADREAD) and breast carcinoma (BRCA) cohorts, respectively (Fig. 4C). Compared to these models, the kidney model exhibited lower performance both within its native cancer types (papillary, clear-cell, and chromophobe renal cell carcinoma; KIRP, KIRC, and KICH) and across non-native entities, indicating limited generalization (Fig. 4C). This is related to the specific morphology of kidney cancer but also to a slightly different initial annotation strategy in this use case (see “Discussion”). The prostate model, while performing well within its native prostate adenocarcinoma (PRAD) cohort and achieving moderate scores in several non-native projects, showed more variable performance across other cancer types (Fig. 4C).

**Table. 1:** Segmentation performance scores across five segmentation models and 21 TCGA projects with row and column means (mean ± SD)

TCGA project	Breast	Colon	Lung	Kidney	Prostate	Row mean ± SD
TCGA-BLCA	7.5 ± 1.6	6.7 ± 2	8.6 ± 1.5	3.5 ± 1.6	5.3 ± 2.2	6.3 ± 2.5
TCGA-BRCA	9.1 ± 0.9	6.6 ± 1.9	7.5 ± 2.2	2.3 ± 1.2	5.8 ± 1.8	6.2 ± 2.8
TCGA-CESC	6.9 ± 1.7	6.6 ± 1.9	9 ± 1.1	2.7 ± 1.1	4.2 ± 1.9	5.9 ± 2.7
TCGA-CHOL	8.5 ± 1.1	7.2 ± 1.3	8.8 ± 1.1	3.3 ± 1.3	6.7 ± 1.9	6.9 ± 2.4
TCGA-COADREAD	7.5 ± 1.2	8.6 ± 1	8.5 ± 1.5	2.7 ± 1	4.8 ± 1.6	6.4 ± 2.6
TCGA-ESCA	7.3 ± 1.4	7.4 ± 1.4	9 ± 1.2	3.6 ± 1.2	4.7 ± 2	6.4 ± 2.5
TCGA-HNSC	5.7 ± 1.8	6.2 ± 1.9	8.6 ± 1.4	2.2 ± 1.1	3 ± 1.7	5.1 ± 2.8
TCGA-KICH	7.6 ± 1.8	5.8 ± 2.2	8 ± 2.1	5.2 ± 1.2	3 ± 2.2	5.9 ± 2.6
TCGA-KIRC	6 ± 2	2.4 ± 2.1	5.2 ± 2.7	4.3 ± 1.5	2.9 ± 2.1	4.2 ± 2.5
TCGA-KIRP	7.3 ± 1.6	5.9 ± 2.1	8 ± 2	4.5 ± 1.4	4.4 ± 2.3	6 ± 2.4
TCGA-LIHC	8 ± 1.4	6.6 ± 1.9	8.5 ± 1.7	3.9 ± 1.2	3.6 ± 2.3	6.1 ± 2.7
TCGA-LUAD	7.4 ± 1.5	7 ± 1.6	9.5 ± 0.9	2.7 ± 1	4.1 ± 1.6	6.1 ± 2.8
TCGA-LUSC	7 ± 1.5	7.1 ± 1.6	9.4 ± 0.9	3.2 ± 1	4.5 ± 1.8	6.2 ± 2.6
TCGA-MESO	7 ± 2.3	5.6 ± 2.2	6.9 ± 2.5	2.8 ± 1.2	4.4 ± 2.5	5.3 ± 2.7
TCGA-OV	8.3 ± 1	7.7 ± 0.9	9.2 ± 0.9	4 ± 1	6.9 ± 1.6	7.2 ± 2.1
TCGA-PAAD	7.5 ± 1.4	6.7 ± 1.6	7.7 ± 1.6	2.6 ± 1.3	4.5 ± 1.9	5.8 ± 2.5
TCGA-PRAD	8.2 ± 1	6.1 ± 2	6.4 ± 1.9	2.1 ± 1.4	7.8 ± 1.5	6.1 ± 2.7
TCGA-SKCM	6.3 ± 2	4.4 ± 2.3	6.2 ± 2.3	2.9 ± 1.4	3.5 ± 2.3	4.7 ± 2.5
TCGA-STAD	7.9 ± 1.3	6.3 ± 2.4	7.4 ± 2.4	2.5 ± 1.1	4.8 ± 2.2	5.8 ± 2.8
TCGA-THCA	8.1 ± 1.1	7.2 ± 1.5	8.8 ± 1.2	3 ± 1.2	6.7 ± 2.1	6.7 ± 2.5
TCGA-UCEC	6.9 ± 1.5	7.5 ± 1.6	8.2 ± 1.5	2.7 ± 1.1	5.1 ± 1.9	6.1 ± 2.5
Column mean ± SD	7.4 ± 1.7	6.4 ± 2.3	7.9 ± 2.1	3 ± 1.4	4.8 ± 2.3	

From a disease entity perspective, certain TCGA cohorts consistently achieved higher segmentation scores across multiple models, indicating better cross-entity generalization. Notably, the ovarian cancer (OV), cholangiocarcinoma (CHOL), and thyroid cancer (THCA) projects were among those with the highest mean scores (7.2 ± 2.1, 6.9 ± 2.4, and 6.7 ± 2.5, respectively), suggesting that these cancer types might show some more typical/generic features present in many other cancer types. On the other hand, cohorts such as KIRC, skin cutaneous melanoma (SKCM), and head and neck squamous cell carcinoma (HNSC) consistently received lower scores (4.2 ± 2.5, 4.7 ± 2.5, and 5.1 ± 2.8, respectively), indicating that these cancer types might show unique morphologies and present unique challenges, at least for some of the tested models (Fig. 4D).

**Fig. 5:**
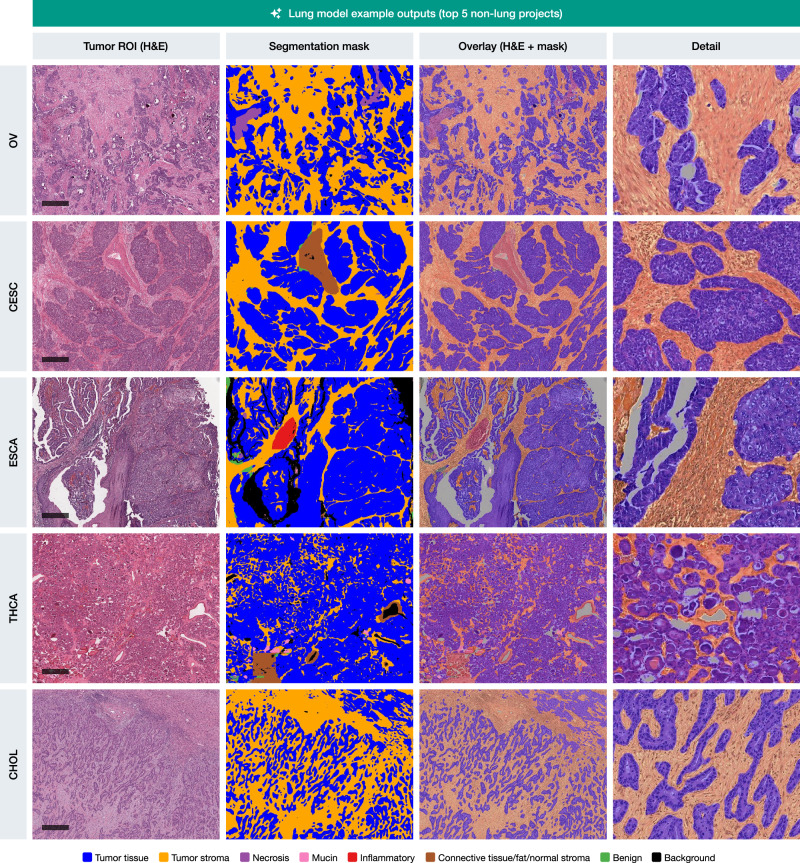
Example outputs from the lung model on the top 5 highest-scoring non-lung TCGA projects. Representative tumor regions of interest (ROIs) from ovarian (OV), cervical (CESC), esophageal (ESCA), thyroid (THCA), and cholangiocarcinoma (CHOL) cancers are shown. For each case, the tumor ROI (H&E staining), the corresponding segmentation mask, an overlay of the H&E image with the segmentation mask, and a detail view providing high-magnification visualization of tumor (blue) versus stroma (yellow) boundary precision are presented. Tumor tissue is accurately differentiated from tumor stroma, demonstrating the model’s strong generalization capabilities across diverse cancer types. Scale bars: 500 μm.

The lung segmentation model demonstrated particularly strong generalization capabilities across several non-lung cancer types, as illustrated in Fig. 5. This figure highlights representative outputs from the top five non-lung TCGA projects—ovarian cancer (OV), cervical squamous cell carcinoma (CESC), esophageal carcinoma (ESCA), thyroid carcinoma (THCA), and cholangiocarcinoma (CHOL), achieving mean scores of up to 9.2 ± 0.9 (OV cohort). In each of these cases, the lung model was able to accurately delineate tumor tissue and tumor stroma regions, with segmentation masks that closely align with the histological features observed in the original H&E images. Supplementary Figs. 2–5 provide similar examples for the other four models, demonstrating their respective top five cross-entity generalization performances.

### Same histological type of divergent localization allows for better generalization

Some histological carcinoma subtypes, e.g., squamous cell carcinomas (SCC), can arise in different locations and even have differing etiologies (e.g., lung SCC and head-and-neck SCC, the latter often associated with HPV in the oropharyngeal region and arising in lymphoepithelial mucosa). On the other hand, some organs develop diffuse adenocarcinomas which have morphological similarities (e.g., diffuse stomach adenocarcinoma and invasive lobular breast carcinoma) and consist of single, cytologically bland discohesive cells, representing a significant challenge for segmentation algorithm training and implementation.

**Fig. 6:**
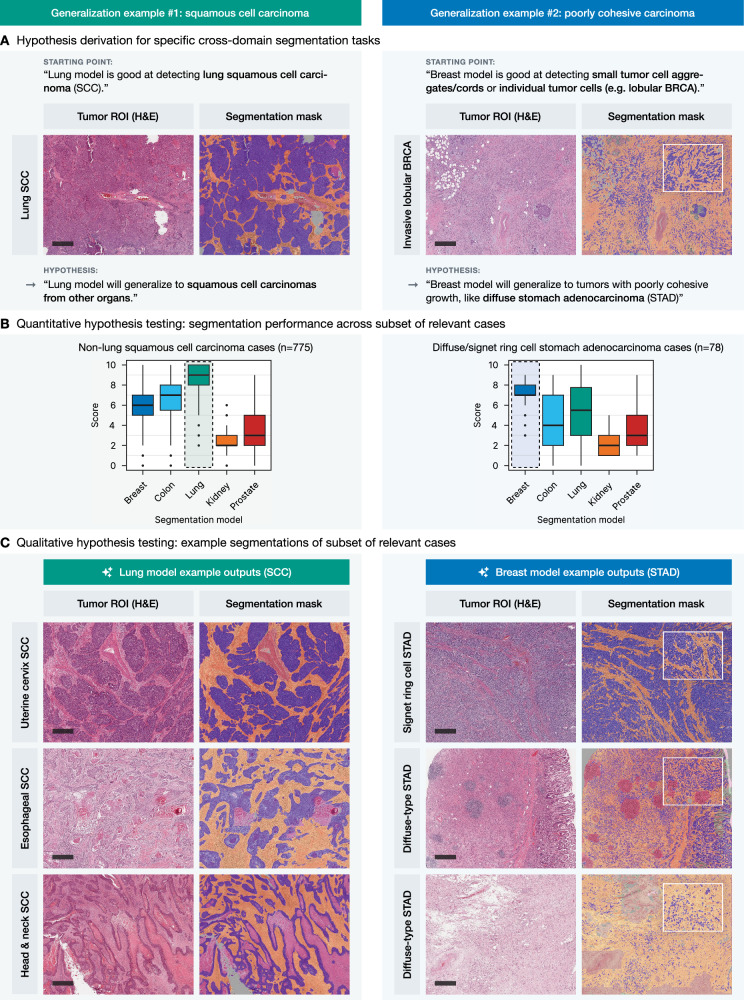
Cross-domain generalization examples for squamous cell carcinoma and poorly cohesive carcinoma. **A** Hypothesis derivation: the lung model, trained on lung squamous cell carcinoma (SCC), is hypothesized to generalize to SCCs from other organs, while the breast model, trained on breast carcinoma including poorly cohesive invasive lobular carcinoma, is hypothesized to generalize to other tumors with poorly cohesive growth patterns, such as diffuse-type stomach adenocarcinoma (STAD). Scale bar: 500 μm. **B** Quantitative analysis: Segmentation performance of the models across relevant subsets of cases. Box plots show model performance for *n* = 775 non-lung SCC cases (left) and *n* = 78 diffuse-type stomach adenocarcinoma cases (right), with the lung model excelling in SCC cases and the breast model excelling in poorly cohesive STAD cases. **C** Example segmentations from the relevant case subsets. The lung model shows effective segmentation of SCCs in uterine cervix, esophageal, and head & neck SCCs, while the breast model accurately segments diffuse-type STAD cases, including signet ring cell and poorly cohesive gastric carcinomas. Scale bars: 500 μm.

In the first use case, the lung segmentation model, known for its strong performance in identifying squamous cell carcinoma (SCC) in lung cancer cases, was applied to SCCs from other organs (*n* = 775; Fig. 6A, left panel). The model demonstrated excellent performance in segmenting SCC cases from the cervix (CESC), esophagus (ESCA), and head and neck (HNSC) regions, as evidenced by high scoring metrics and accurate delineation of tumor structures, compared to the other tested models (Fig. 6B, C, left panel). In the second use case, the breast segmentation model, trained to detect small tumor cell aggregates and individual tumor cells in cases of invasive lobular carcinoma as part of the initial development, was tested in cases with diffuse-type gastric adenocarcinoma (*n* = 78; Fig. 6A, right panel). Again, quantitative and qualitative analysis showed that the breast model efficiently segmented small, dispersed tumor cell clusters in diffuse-type or signet-ring cell gastric adenocarcinoma (STAD) cases with very good to excellent semiquantitative scores of 7–9 (Fig. 6B, C, right panel). Supplementary Fig. 7 provides a detailed breakdown of the non-lung SCC cases and diffuse-type carcinoma cases included in this analysis by specific diagnosis.

### Cross-domain generalization for accurate benign/precusor tissue structure segmentation

**Fig. 7:**
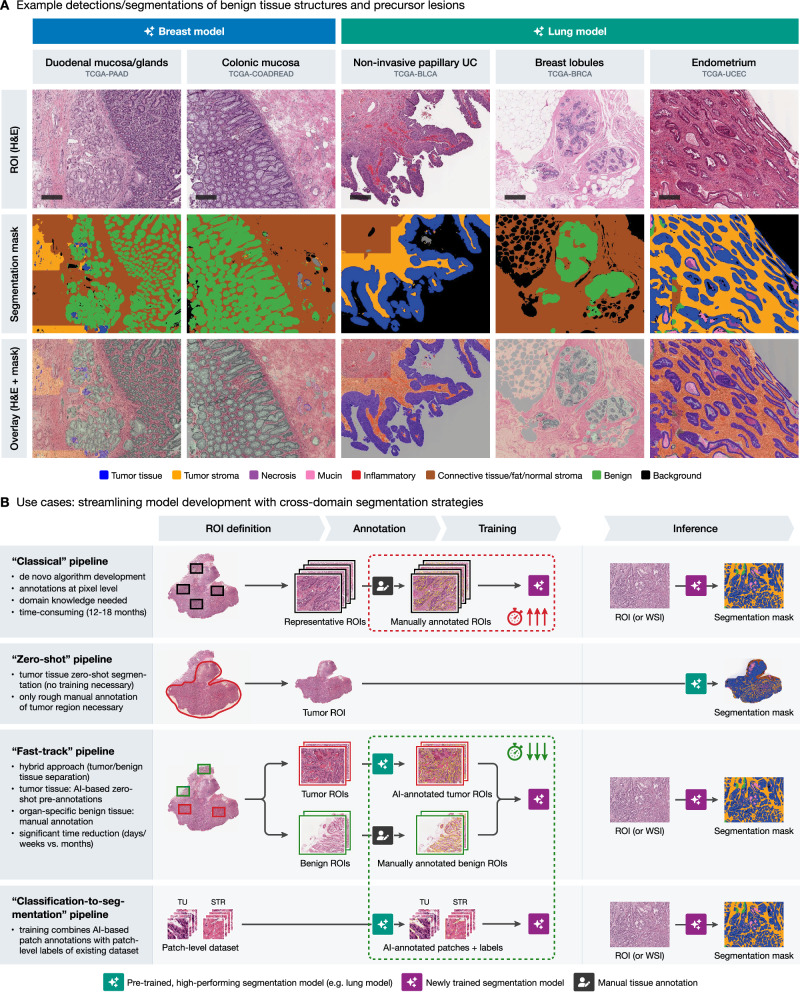
Detection and segmentation of benign/precusor tissue structures and proposed strategies for streamlining model development. **A** Example segmentations of benign tissue structures and precursor lesions by the breast and lung models. The breast model effectively segments glandular tissues such as duodenal mucosa (PAAD cohort, pancreatic adenocarcinoma) and colonic mucosa (COADREAD cohort, colonic/rectal adenocarcinoma), while the lung model accurately identifies non-invasive papillary urothelial carcinoma (UC; BLCA cohort, bladder carcinoma), benign breast lobules (BRCA cohort, breast cancer), and endometrium (UCEC cohort, endometrial carcinoma). Depending on the model, structures are classified as benign tissue (green) or tumor tissue (blue)—these outputs can still be used for automatic annotations (Fig. 6B) and can be re-labeled during training iterations. Scale bars: 500 μm. **B** Use cases for cross-domain segmentation strategies to streamline model development. The classical pipeline involves de novo model development with extensive manual annotations, requiring significant time and domain knowledge. The “zero-shot” pipeline leverages pre-trained models like the lung model for immediate segmentation of tumor regions of interest (ROIs) with no further manual annotation. The “fast-track” pipeline combines AI-based pre-annotations for tumor regions with manual annotation of benign tissues, significantly reducing the time required for model development. The “classification-to-segmentation” pipeline leverages existing patch-level classification datasets and can convert them into pixel-level segmentation datasets using their patch-level labels. TU tumor, STR stroma.

Beyond the primary goal of tumor region segmentation, we also examined the performance of the models on benign tissue regions, which were annotated alongside the tumor ROIs (as shown in Fig. 1). Two experienced pathologists assessed the regions segmented by all models and provided a qualitative assessment whether the models could identify meaningful structures in these regions (“off-label” effects were also considered, e.g., when benign structures were detected as tumor but were well-segmented as this can also be effectively used for tasks such as gland segmentation etc.). Figure 7A summarizes the review results and provides representative examples. Here, the breast model accurately segmented glandular tissues such as duodenal mucosa and submucosal glands or colonic mucosa. Similarly, the lung model reliably detected benign breast lobules, as well as other benign structures such as endometrium, across various TCGA projects. Moreover, non-invasive dysplastic/tumorous lesions can be also processed with high accuracy by the models, e.g., non-invasive papillary urothelial carcinoma (Fig. 7A).

### Cross-domain generalization-based strategies offer fast-track alternatives to classical model development

Based on the presented findings, there is a large potential for leveraging cross-domain generalization capabilities of previously developed models in training new tools. Figure 7B illustrates three potential strategies to streamline the development of new segmentation models, compared to a “classical” approach. The latter typically involves extensive manual de novo multi-class annotations of representative tumor and benign tissue regions at the pixel level by a domain expert, usually a pathologist, followed by model training and validation as well as numerous iteration steps for model optimization. This is a costly and time-consuming process that can take up to 12–18 months. In contrast, a “zero shot” pipeline can leverage a high-performing segmentation model, such as the lung model, by directly applying it to a roughly annotated tumor ROI, eliminating the need for training a new model entirely. This zero-shot approach takes advantage of the model’s generalization capabilities, providing immediate segmentation outputs useful for exploratory research or preliminary analyses without additional training. A “fast-track” pipeline further optimizes the process by combining zero-shot segmentation of tumor and benign ROIs to produce precise pre-annotations that need only minimal corrections by experts. These annotations are then used to train a new model for a new malignant tumor type. This hybrid strategy significantly reduces the annotation burden and has been demonstrated to accelerate model development, potentially reducing the time required to less than 7 days.^25^ In addition, there is an abundance of valuable open-source pathology datasets for classification tasks (patch-level ground truth). These datasets can be transformed to full pixel-wise segmentation datasets leveraging available patch-level labels and processing by the available developed models.^23^ The best model (organ) selection for all mentioned approaches can be effectively based on the provided analysis of cross-domain generalization (Figs. 4–7).

## Discussion

Our study addresses two important bottlenecks in the development of computational pathology models for malignant tumors. First, developing an accurate, clinical-grade tool requires a substantial time investment for preparing a training dataset, sometimes exceeding 12 months of annotation work by domain experts (pathologists), followed by multiple iterative cycles of inclusion of additional cases, post-annotation, and algorithm re-training.^14,22,24^ Second, the current development paradigm predominantly focuses on training algorithms for single tumor types and tasks. Although some effective multi-task learning methods exist,^34,35^ most algorithms still remain limited to single tumor entities. In our previous studies, we were able to develop highly precise new models using fast-track principles and *intra-domain* generalization, e.g., fully automatic pre-annotations of unseen data for non-small cell lung cancer subtyping model development,^14^ development of lymph node metastasis detection tool for colorectal cancer leveraging a primary colorectal cancer model,^25^ and transforming a classification task training dataset into a precise pixel-wise segmentation dataset for a clinical-grade tool for prostate specimen processing.^23^

In this study, we systematically investigate *cross-tumor* domain generalization in a large dataset of 21 malignant tumors (*n* = 20 epithelial tumors and malignant melanoma) for 5 previously developed mono-tumor models (lung, prostate, colorectal, breast, and kidney; Fig. 1. Notably, based on our developed software (Supplementary Fig. 1) and evaluation pipeline (Fig. 1B), we demonstrate the ability of these models to accurately segment tumor tissue into relevant classes (epithelial tumor compartments and tumor associated classes, i.e., tumor stroma, necrotic debris, and mucin) in various cancer entities beyond their initial training domains, offering significant potential for more efficient development of computational pathology tools (Fig. 4A–D).

The lung model emerged as particularly robust, achieving high segmentation scores not only in its native lung cancer cohorts but also across a wide range of other cancer types. It reached excellent segmentation quality (scores 8–10) for 11/19 other malignant tumors, with very good quality (score 7) in an additional 5/19 entities (Fig. 4D). In comparison, the breast model achieved excellent generalization in 4/20 tumors (good in 13/20), the colorectal model in 0/20 (good in 7/20), and the prostate model in 0/20 (good in 3/20) tumors. For reference, all models were also tested on their native domains (Fig. 4C, D). Notably, an “excellent” score (8–10) indicates that the segmentation quality for non-native tumors matches that of the model’s native domain, demonstrating *remarkable* generalization capability. A “very good” quality score still suggests a very high performance, however, some imprecisions might occur (e.g., by outlier cases) and should be accounted for.

The lung model’s training on both lung adenocarcinoma and lung squamous cell carcinoma likely contributed to its strong performance in other squamous cell carcinomas, such as those found in the esophagus, head-and-neck area, and cervix (Fig. 6A–C). Additionally, its ability to recognize the varied morphologies of lung adenocarcinoma—including glandular, papillary, and solid growth patterns—enabled it to generalize effectively to other adenocarcinomas with similar histological features, such as adenocarcinomas of the ovary, esophagus, and bile ducts or thyroid carcinoma. Similarly, the breast model’s strength lies in its ability to segment diverse morphologies, including the gland-forming patterns seen in the more prevalent invasive breast carcinoma subtypes such as the so-called ductal carcinoma (or carcinoma of no special type) and, on the other hand, the poorly cohesive growth patterns characteristic of invasive lobular carcinoma. This unique capability makes this model particularly effective in generalizing to other cancers with similar morphologies, such as diffuse gastric carcinoma, as explored in Fig. 6A–C.

In contrast, the prostate model, while excellent at detecting prostate adenocarcinoma, shows more limited generalization. Acinar adenocarcinoma, which constitutes 95% of prostate cancers, is characterized by atypical glandular structures. Given that this model was primarily trained on recognizing these glandular patterns, it is less likely to perform well in detecting morphological structures less prevalent in prostate cancer, such as squamous, papillary, or solid differentiation. This limitation reflects the model’s specialization in a more homogeneous tumor type. Certain cancer entities, such as clear cell renal cell carcinoma (KIRC), melanoma (SKCM), and partially mesothelioma (MESO), consistently posed challenges for the models. KIRC, characterized by tumor cells with optically clear cytoplasm, presents a distinct morphology that is not commonly found in the entities the models were trained on, leading to inconsistent performance. The variability within KIRC, where higher-grade tumors can exhibit more recognizable eosinophilic cytoplasm, could explain the high standard deviation observed in the lung model’s performance in this cohort (5.2 ± 2.7). Similarly, SKCM and MESO are known for their morphological diversity and outlier histology compared to carcinomas, with both entities often displaying spindle-cell morphology and varying pigmentation in the case of melanoma. These ‘chameleon-like’ characteristics, which also challenge human pathologists, likely contribute to the models’ difficulty in generalizing to these tumor types, resulting in lower and more variable performance. SKCM and MESO tumors showing epitheloid morphology were segmented with a quality comparable to that of carcinomas. Additionally, our study primarily focused on epithelial tumors which aligns with the models’ training on carcinomas. We excluded other tumor entities such as soft tissue and bone tumors (sarcomas), neoplasms of the nervous system, germ cell tumors, and hematological neoplasms. The limited generalization observed in melanoma and mesothelioma underscores the challenges of extending these models to tumor types beyond epithelial origins.

To our knowledge, our study offers the first systematic evaluation of pan-tumor generalization for segmentation models across a wide range of malignancies, demonstrating the feasibility of applying models trained on a single tumor type to multiple distinct cancer entities. This cross-domain capability holds significant promise for reducing the time and resources needed for the development of new, tumor-specific segmentation models, while also paving the way for the future creation of pan-cancer models with precise pixel-level segmentation across all tumor types. We propose innovative strategies for accelerating the development of new computational pathology tools (Fig. 7). Traditionally, training segmentation models de novo is a time-consuming process, requiring extensive manual annotations and numerous iterations for model refinement. However, our findings indicate that models with strong cross-entity generalization capabilities, such as the lung model, can be leveraged either through zero-shot segmentation or in a “fast-track” approach. We have previously demonstrated the effectiveness of this fast-track strategy in reducing the annotation step to approximately one week by applying the existing colon model to a lymph node detection task in colorectal cancer.^25^ Additionally, we have recently published a study where we refined existing region-level annotations into a precise prostate segmentation algorithm, reducing the time needed for manual annotations by around 80%.^23^

In the broader context of computational pathology, the shift toward weakly supervised approaches for tasks such as tumor subtyping and pan-tumor detection has gained significant traction. Recently released foundation models (e.g., UNI, Virchow, Prov-GigaPath^6,9,10^) act as feature extractors for histopathology but are limited by their 224 px patch size. This technical constraint restricts their direct applicability to multi-class semantic segmentation. Pixel-level predictions at patch boundaries (approximately 64 pixels from each edge) are inherently less reliable, and with 224 px patches, the regions with reliable predictions become very small. Furthermore, established segmentation architectures such as UNet and UNet++ are designed for power-of-two patch sizes (256, 512, 1024 px) to allow multiple downsampling steps; using 224 px patches would require architectural modifications or result in information loss. These constraints explain why foundation model publications benchmark single-cell segmentation and classification tasks—where small patch sizes are appropriate—but not multi-class tissue semantic segmentation. Additionally, our recent work^14^ shows that these models combined with weakly supervised learning perform poorly on biopsy samples and learn many distracting statistical biases from images (e.g., assigning high attention scores to benign tissue such as necrosis or cartilage), with our fully supervised pixel-wise segmentation approach outperforming both UNI and Prov-GigaPath foundation encoders on patch-level classification, and showing particularly marked superiority in biopsy specimens where foundation model-based approaches exhibited significant accuracy drops. This highlights the need for fully supervised methods, which offer greater flexibility in fine-tuning for problematic regions and class balancing in the setting of non-resection specimens. Evidence from multiple studies demonstrates that foundation models show particular advantages in label efficiency and few-shot learning scenarios.^6^^,^^8^^,^^9^ With the large, densely annotated training datasets used for our models (>200–300 whole-slide images each with pixel-level annotations), the expected advantage of foundation encoders over ImageNet-pretrained encoders is likely diminished—though this remains an empirical question for semantic segmentation tasks specifically. Other studies, like that by Noorbakhsh et al.,^36^ show that models trained on single tumor types can cross-classify tumors across different entities, indicating shared morphological features. However, they focus on classification rather than segmentation. Our study builds on this, extending cross-entity generalization to precise tumor segmentation. The future goal is to develop a pan-cancer segmentation approach capable of pixel-level accuracy, unlike existing pan-cancer models that focus on tumor presence or molecular predictions at a coarser spatial resolution.^37–40^ Fully supervised methods remain essential for achieving the spatial precision and explainability required for clinical applications.

Our study is not devoid of limitations. Direct comparisons between the models should be interpreted with caution, as technical differences in their development significantly impact performance. The lung model, for example, benefited from extensive, high-resolution annotations, enabling it to distinguish tumor tissue from stroma with great precision, even in small regions like those between individual tumor glands. In contrast, the kidney model was trained with fewer and less detailed annotations, resulting in reduced segmentation resolution. These differences help explain why the lung model performs on par with the colon model in the colon-native COADREAD cohort and even outperforms the kidney model in the kidney-native KICH, KIRC, and KIRP cohorts. Our study uses TCGA data for both model training and evaluation, which warrants careful interpretation. Each TCGA project comprises cases from up to 36 different pathology institutions with varying laboratory practices—including differences in fixation, sectioning, and staining protocols—as well as different digitization equipment and artifact patterns. This heterogeneity means that TCGA effectively functions as a multi-institutional cohort rather than a single homogeneous dataset. Furthermore, for most models, only 20–30% of available images from the respective training cohorts were used. It should be noted that same-domain evaluations are inherently biased since models were trained on subsets of the same TCGA datasets used for evaluation. However, we consider this advantageous as it establishes a stringent benchmark for cross-domain performance. Our primary focus is cross-domain generalization to entirely different TCGA projects, which involves completely independent cases with no overlap between training and test data. We use same-domain results only as comparative references. Remarkably, several cross-domain evaluations achieved performance virtually identical to same-domain results, demonstrating robust generalization despite these stringent conditions.

In addition to these technical considerations, our semiquantitative scoring system remains a subjective measure reliant on manual assessment by pathologists, introducing some variability. To address this concern, we conducted comprehensive validation studies (Fig. 3) demonstrating excellent inter-rater reliability (ICC = 0.87) and strong concordance between manual scores and objective Dice coefficients against available ground truth annotations. This validation supports the robustness of our scoring methodology despite its inherent subjectivity and demonstrates that our approach effectively captures segmentation performance for large-scale cross-domain analysis. Despite this limitation, our systematic approach—applied to a large and diverse cohort—provides valuable insights and represents a significant step beyond anecdotal evidence in this area of research. The generalization capabilities observed in our study are not confined to the specific models we developed. Our findings are based on five distinct models trained on different cancer types, and the consistent performance across multiple, unrelated tumor entities suggests that this approach has significant potential for broader application. Our results suggest that models with robust training datasets and diverse morphological exposure (like our lung model) may have enhanced cross-cancer capabilities, but we recognize that generalization performance will likely depend on factors such as training data quality, annotation strategies, model architecture, and the morphological diversity within training cohorts. Additionally, by using a large, diverse dataset from TCGA, our study ensures that the results are representative of a wide range of cancer morphologies within the scope of our analysis. Our work establishes a systematic framework for evaluating cross-cancer generalization and provides evidence supporting this approach, while acknowledging that each new model-cancer combination should be validated rather than assumed to generalize based solely on our findings. This represents a significant step beyond anecdotal evidence in demonstrating the feasibility and potential of cross-domain generalization in computational pathology.

## Conclusions

Our systematic evaluation of five segmentation models across 21 cancer types demonstrates that cross-domain generalization is feasible, with the lung model achieving excellent segmentation quality in 11 of 19 non-native tumor entities. Morphological similarity—shared histological subtypes or growth patterns—emerges as a key determinant of successful transfer, enabling development strategies that can reduce model training time from 12 to 18 months to under one week. This study represents an important step toward the idea of pan-cancer foundational segmentation models, demonstrating the potential of cross-entity generalization in AI-driven pathology. By enabling precise, high-resolution segmentation across multiple tumor types, our models pave the way for more efficient diagnostic tools and reproducible biomarker discovery. Future work will focus on expanding these capabilities to create true pan-cancer segmentation models, which will integrate with emerging technologies such as single-cell analysis and spatial transcriptomics to further enhance our understanding of tumor heterogeneity and the tumor microenvironment.

## Supplementary information

Supplementary Material

## Data Availability

Whole-slide images used in this study are publicly available from The Cancer Genome Atlas (TCGA) via the Genomic Data Commons (https://portal.gdc.cancer.gov/). Source data underlying all figures, including segmentation quality scores, Dice coefficients, inter-rater reliability data, and clinical metadata, are deposited on Zenodo (https://zenodo.org/records/18518811). Tumor tissue ROIs extracted from all 21 TCGA cohorts are deposited on Zenodo (https://zenodo.org/records/18668580). Segmentation masks for all model predictions are deposited on Zenodo (https://zenodo.org/records/18669667) under an embargo until February 1, 2027; the dataset will become freely available automatically after this date. All custom code is available on GitHub (https://github.com/tbedau/cross-cancer-segmentation) and archived on Zenodo (https://zenodo.org/records/18520078).

## Code availability

All custom code used in this study, including QuPath annotation scripts, the segmentation inference pipeline, the browser-based scoring application, and the R/Quarto analysis notebooks used to generate all figures and statistical analyses, is available on GitHub (https://github.com/tbedau/cross-cancer-segmentation) and archived on Zenodo (https://zenodo.org/records/18520078).^41^ The repository also includes a data retrieval script that automatically downloads the source data from the Zenodo archives listed above. All Python and R dependencies are version-locked via uv and renv lock files in the repository for full reproducibility.
