# Supplementary material for: Comprehensive evaluation of cross cancer generalization in histopathology segmentation models across 21 tumor types
Source: Commun Med (Lond). 2026 May 5;6:302. doi: 10.1038/s43856-026-01601-x (PMC13195172; doi:10.1038/s43856-026-01601-x)
Supplement: Supplementary file 1 — Supplementary Material [file 43856_2026_1601_MOESM1_ESM.docx]

# Supplementary Figure 1

Scoring application architecture and interface. (A) The architecture of the scoring app includes a client-side HTMX web app that interacts with a server based on FastAPI (Python). The server manages the scoring data stored in an SQLite database and handles image data in Deep Zoom Image (DZI) format. Users input scores through the web interface, and the data can be exported as a CSV file. (B) Project overview interface, displaying a list of TCGA projects with their respective statuses, allowing users to track and manage scoring progress. (C) Individual project view, presenting a detailed list of cases within a selected TCGA project, including the segmentation scores for each model. (D) Sample view from the scoring app, showing side-by-side comparisons of segmentation outputs from different models, allowing users to score the accuracy of tumor/stroma segmentation directly within the interface. Images can be zoomed and panned using OpenSeadragon for detailed examination if necessary.

# Supplementary Figure 2

Example outputs from the breast model on the top 5 highest-scoring non-breast TCGA projects. Representative tumor regions of interest (ROIs) from cholangiocarcinoma (CHOL), ovarian (OV), prostate (PRAD), thyroid (THCA), and liver hepatocellular carcinoma (LIHC) cancers are shown. For each case, the tumor ROI (H&E staining), the corresponding segmentation mask, an overlay of the H&E image with the segmentation mask, and a detail view providing high-magnification visualization of tumor (blue) versus stroma (yellow) boundary precision are presented. Scale bars: 500 μm.

# Supplementary Figure 3

Example outputs from the colon model on the top 5 highest-scoring non-colon TCGA projects. Representative tumor regions of interest (ROIs) from ovarian (OV), uterine corpus endometrial carcinoma (UCEC), esophageal (ESCA), thyroid (THCA), and cholangiocarcinoma (CHOL) cancers are shown. For each case, the tumor ROI (H&E staining), the corresponding segmentation mask, an overlay of the H&E image with the segmentation mask, and a detail view providing high-magnification visualization of tumor (blue) versus stroma (yellow) boundary precision are presented. Scale bars: 500 μm.

# Supplementary Figure 4

Example outputs from the kidney model on the top 5 highest-scoring non-kidney TCGA projects. Representative tumor regions of interest (ROIs) from ovarian (OV), liver hepatocellular carcinoma (LIHC), esophageal (ESCA), bladder (BLCA), and cholangiocarcinoma (CHOL) cancers are shown. For each case, the tumor ROI (H&E staining), the corresponding segmentation mask, an overlay of the H&E image with the segmentation mask, and a detail view providing high-magnification visualization of tumor (blue) versus stroma (yellow) boundary precision are presented. Scale bars: 500 μm.

# Supplementary Figure 5

Example outputs from the prostate model on the top 5 highest-scoring non-prostate TCGA projects. Representative tumor regions of interest (ROIs) from ovarian (OV), cholangiocarcinoma (CHOL), thyroid carcinoma (THCA), breast carcinoma (BRCA), and bladder carcinoma (BLCA) are shown. For each case, the tumor ROI (H&E staining), the corresponding segmentation mask, an overlay of the H&E image with the segmentation mask, and a detail view providing high-magnification visualization of tumor (blue) versus stroma (yellow) boundary precision are presented. Scale bars: 500 μm.

# Supplementary Figure 6

Diagnosis breakdown of cases included in the analysis for Figure 5B. (A) Distribution of non-lung squamous cell carcinoma cases across different TCGA projects. (B) Distribution of diffuse-type carcinoma cases.

# Supplementary Table 1

Assignment of colors to various tissue classes for different cancer segmentation models.

|  | Breast | Colon | Lung | Kidney | Prostate |
| --- | --- | --- | --- | --- | --- |
| Blue | TUMOR, DCIS, LCIS | TUMOR, ADENOM_HG | TUMOR | TUMOR | TUMOR |
| Yellow | TUMOR STROMA | TU_STROMA | TUMOR STROMA | STROMA | N_STR |
| Purple | NECROSIS | ULCUS, NECROSIS | NECROSIS | NECROSIS |  |
| Pink | MUCIN | MUCIN | MUCIN |  |  |
| Red | INFLAM | LYMPH_NODE, LYMPH_TIS, LYM_AGGR | LYMPH_AGGR, LYMPH_NODE |  |  |
| Brown | FAT, STROMA | ADVENT, VESSEL | STROMA, NERVE, FAT, MUSCLE, VESSEL | FAT |  |
| Gray | BLOOD | BLOOD | BLOOD | TUMOR_REGRESS, BLOOD |  |
| Green | SKIN, BEN EPIT, SKIN ADNEX | MUC, ADENOM_LG, SUBMUC, MUSC_PROP, MUSC_MUC | BENIGN LUNG, BRONCHUS, CARTILAGE, GLAND_BRONCH | KIDNEY_BENIGN, UROTHEL, ADRENAL | N |
| White | BACK | BACK | BACK | BACK | BACK |
